# d Rhamnose β-hederin reverses chemoresistance of breast cancer cells by regulating exosome-mediated resistance transmission

**DOI:** 10.1042/BSR20180110

**Published:** 2018-09-28

**Authors:** Wei-xian Chen, Ling-yun Xu, Qi Qian, Xiao He, Wen-ting Peng, Wen-qiang Fan, Yu-lan Zhu, Jin-hai Tang, Lin Cheng

**Affiliations:** 1 Department of Breast Surgery, The Affiliated Changzhou No. 2 People’s Hospital of Nanjing Medical University, Changzhou, China; 2 Department of Breast Surgery, The First Affiliated Hospital of Nanjing Medical University, Nanjing, China

**Keywords:** Breast cancer, Chemoresistance, D Rhamnose β-Hederin, Exosomes

## Abstract

d Rhamnose β-hederin (DRβ-H), an active component extracted from the traditional Chinese medicinal plant *Clematis ganpiniana*, has been reported to be effective against breast cancer. Recent studies have also indicated that the isolated exosomes (D/exo) from docetaxel-resistant breast cancer cells MCF-7 (MCF-7/Doc) were associated with resistance transmission by delivering genetic cargo. However, the relevance of D/exo during DRβ-H exposure remains largely unclear. In the present work, exosomes were characterized by morphology and size distribution. We reinforced the significant role of D/exo in spreading chemoresistance from MCF-7/Doc to recipient sensitive cells after absorption and internalization. DRβ-H could reduce the formation and release of D/exo. Next, we demonstrated that DRβ-H was able to reverse docetaxel resistance and that D/exo was responsible for DRβ-H-mediated resistance reversal. We also found that DRβ-H could decrease the expressions of several most abundant miRNAs (*miR-16, miR-23a, miR-24, miR-26a*, and *miR-27a*) transported by D/exo. Target gene prediction and pathway analysis showed the involvement of these selected miRNAs in pathways related to treatment failure. Our results suggested that DRβ-H could reduce D/exo secretion from MCF-7/Doc cells and induce the reduction in resistance transmission via D/exo.

## Introduction

Breast cancer is the most common malignant tumor in women worldwide [1]. Although docetaxel plays an important role in first-line chemotherapy, drug resistance remains a major obstacle to successful treatment of breast cancer and leads to poor overall survival for patients. The mechanisms of chemoresistance are under intense research, and much attention has been recently paid to the intercellular transfer of tumor-derived exosomes as vehicles for genetic cargo [2,3].

Exosomes are nanosized vesicles of endocytic origin secreted by most cell types and contain a wide variety of active molecules including proteins, lipids, mRNAs, and miRNAs. They could be shuttled into tumor microenvironment and then absorbed by surrounding cancer cells or stromal cells to release their contents, thus serving as mediators for cell-to-cell communication and a potential mechanism for drug resistance [3–5]. Such phenomenon has been reported in several tumor models, including prostate cancer, glioblastoma, and hepatocellular cancer [6–8]. By using the ready-established cell lines, our previous study also found that docetaxel-resistant breast cancer cells could spread chemoresistance to sensitive cells by shedding abundant exosomes and that the effects could be partly attributed to the constant transfer of specific miRNAs [9]. Given that genetically engineered exosomes carrying therapeutic mRNAs/proteins still have limitations on clinical application, reducing the formation and shedding of tumor-derived exosomes may be a promising strategy to restore chemosensitivity [10,11].

d Rhamnose β-hederin (3β-[(α-l-arabinopyranosyl)-oxy] olean-12-en-28-oicacid) (DRβ-H) is an active component isolated from the roots and rhizomes of the Chinese natural plant *Clematis ganpiniana* [12]*.* DRβ-H has been previously reported to inhibit growth and induce apoptosis of breast cancer cells. In the previous work, our group demonstrated that DRβ-H induced mitochondria-mediated apoptosis, regulated expression of Bcl-2 family proteins, and induced apoptosis by inhibiting PI3K/Akt signaling cascade; and activating ERK pathway [13]. Moreover, we confirmed that DRβ-H could inhibit migration and invasion of human breast cancer cell line [14]. However, the relevance of exosomes and contained miRNAs during DRβ-H exposure remains largely unclear.

Since reduced apoptosis, elevated proliferation, and enhanced invasiveness are all hallmarks of drug resistance [15], it implies that DRβ-H could be a novel candidate for the breast cancer treatment, and might reverse chemoresistance. Therefore, the aim of the present study was to evaluate whether DRβ-H acts through regulating the formation and secretion of exosomes and whether this mechanism is responsible for resistance reversal effect.

## Materials and methods

### Cell culture and drug preparation

The cell lines used here were drug-sensitive MCF-7 breast cancer cells (MCF-7/S) purchased from the Cell Bank of the Chinese Academy of Sciences (Shanghai, China) and a docetaxel-resistant variant of MCF-7 cells (MCF-7/Doc) established in our laboratory as recently described. They have been validated earlier by our group as appropriate models for investigating chemotherapy failure *in vitro* [16]. In selected experiments, MCF-7/S expressing GFP (GFP-S) was generated as previously reported [17]. Cells were incubated in an atmosphere of 5% CO_2_ at 37°C and fed with Dulbecco’s modified Eagle’s medium (DMEM) high glucose (HyClone, U.S.A.), supplemented with 10% FBS, 100 U/ml penicillin, and 100 μg/ml streptomycin. All the cell lines have been confirmed to be free of mycoplasma by using the Mycoplasma Detection Kit-Quick Test (Biotool, U.S.A.). Exosome-depleted FBS was prepared by ultracentrifugation (Avanti J-30I, Beckman Coulter, U.S.A.) at 100000 ***g*** and used for all studies. Protocols of the extraction, purification, and analysis of DRβ-H were described in our recent work [12].

### Exosome isolation and characterization

Exosomes were obtained from the supernatants of MCF-7/S and MCF-7/Doc using a series of centrifugation and ultracentrifugation steps and respectively named as S/exo and D/exo for simplicity. Briefly, the medium was sequentially centrifuged at 300 ***g*** for 10 min, 2000 ***g*** for 15 min, and 12000 ***g*** for 30 min, followed by filtering of the resulting supernatants through a 0.22-μm filter. The filtrates were further ultracentrifuged at 100000 ***g***(Avanti J-30I, Beckman Coulter, U.S.A.) for 2 h, and the exosome pellets were washed in PBS. They were lysed for protein/RNA extraction, labeled for confocal observation, and diluted for incubation assay. In selected experiments, D/exo were treated with 5 U/ml RNase (Ambion, U.S.A.) for 3 h at 37°C, the reaction was ended by 10 U/ml RNase inhibitor (Ambion, U.S.A.) followed by ultracentrifugation. The RNase-treated D/exo were designated as RNase D/exo. The morphology and size of exosomes were observed by TEM as we previously reported [17]. Briefly, exosome pellets were added on parafilm and covered with a 300-mesh copper grid for 45 min. After washing in PBS, the copper grid was fixed in 3% glutaraldehyde, washed, and then contrasted in 2% uranyl acetate. Images were obtained on TEM (JEM-1010, JEOL, Japan) at an acceleration voltage of 80 kV.

### Exosome uptake

Exosomes were labeled with the PKH26 Red Fluorescent Cell Linker Kit (Sigma–Aldrich, U.S.A.) in accordance with the manufacturer’s instructions. As the negative control, exosomes without PKH26 staining were prepared. Uptake of exosomes was studied on GFP-S (1 × 10^5^ cells per well) seeded in six-well plates. After 3 days of incubation, GFP-S cells were cultured with 80 μg PKH-26 labeled exosomes for 24 h. Then they were visualized by a confocal laser scanning microscope LSM710 (Carl Zeiss, Germany) as reported in our publications [17].

### Apoptosis assay

When cells seeded in six-well plates had attached, the supernatants of MCF-7/S were removed and fresh media containing vehicle, S/exo, and D/exo were added. Following a 72-h incubation, cells were treated with 50 nM docetaxel for 24 h. Then, apoptotic rates of MCF-7/S subgroups were evaluated using the Annexin-V-FITC Apoptosis Detection Kit (BD Biosciences, U.S.A.) according to the manufacturer’s protocols and analyzed by flow cytometry (BD FACSCalibur, U.S.A.) as previously documented [13]. MCF-7/Doc cells and MCF-7/Doc subgroup incubated with D/exo were maintained under the same conditions as controls.

### MTT assay

Five groups of cells, namely, MCF-7/S+vehicle, MCF-7/S+S/exo, MCF-7/S+D/exo, MCF-7/Doc, and MCF-7/Doc+D/exo were prepared as described above and cultured in 96-well plates for 24 h. Then, serial dilutions of docetaxel were added. Two days later, 20 μl MTT (Sigma, Germany) was added into each well at 5 mg/ml final concentration for 4 h. After removal of the culture medium, 150 μl DMSO was added for 20 min. The absorbance at 550 nm was detected by CliniBio 128 (ASYS-Hitech, Austria), and the IC_50_ was calculated by SPSS 20.0 package.

### Co-culture assay

GFP-S cells were cultured with MCF-7/Doc in 1:1 ratio in six-well plates, and the samples of cell mixture were divided into four groups. After cells had attached, the supernatants were removed, and fresh media containing vehicle, DRβ-H, DRβ-H+D/exo, and DRβ-H+RNase D/exo were added for 48 h. Then, total GFP-S number was microscopically recorded (ImagingA1, Carl Zeiss, Germany) in three non-consecutive fields (magnification ×200). After a 24-h exposure to 50 nM docetaxel, floating cells were discarded, and residual GFP-S cells were counted under fluorescence microscope.

To evaluate the effects of DRβ-H on apoptosis, cell mixtures treated for 48 h with vehicle, DRβ-H, DRβ-H+D/exo, and DRβ-H+RNase D/exo were prepared. After 24 h exposure to 50 nM docetaxel, apoptotic rates of GFP-S were evaluated using the Annexin-V-FITC Apoptosis Detection Kit (BD Biosciences, U.S.A.) based on the manufacturer’s instructions and then analyzed by flow cytometry (BD FACSCalibur, U.S.A.).

### Western blot

Exosomes were lysed using the Total Exosome RNA and Protein Isolation Kit (Invitrogen, U.S.A.) in accordance with the manufacturer’s instructions, and exosomal proteins were extracted for Western blot. Proteins were electrophoresed through SDS/PAGE gel and transferred to PVDF membranes. CD63 protein level was quantitated using antibody against CD63 (Santa Cruz Biotechnology, U.S.A.). β-actin (Sigma, Germany) was used to normalize for protein loading. Bound proteins were visualized by the ECL Plus Kit (Millipore, U.S.A.) with Image Lab Software (Bio-Rad, U.S.A.).

### RNA isolation and PCR analysis

Exosomal RNA was isolated using the Total Exosome RNA and Protein Isolation Kit (Invitrogen, U.S.A.) in accordance with the manufacturer’s protocols. In selected experiment, cellular RNA was extracted using the mirVana RNA Isolation Kit (Ambion, U.S.A.) in accordance with the manufacturer’s protocols after a 48-h incubation with D/exo, S/exo, and vehicle in six-well plates. Expressions of *Sprouty2, p27*, and *MMP-2* were evaluated. The real-time PCR was performed using SYBR green technique. Briefly, cDNA for miRNA was synthesized using the BU-Script RT Kit (Biouniquer Technology, Nanjing, China) on an iCycler iQ system (Bio-Rad, U.S.A.), and specific stem-loop primers (Springen Biotechnology, Nanjing, China) were designed for the selected miRNAs. All reactions, including the no-template controls, were run in triplicate on a Light Cycler 480 (Roche, Australia). The relative miRNA expressions were calculated using ΔΔ*C*_t_ method and normalized to *U6*.

### Evaluation of miRNA transfer

GFP-S cells seeded in six-well plates were harvested after 4, 12, and 24 h incubation with evenly divided D/exo or without D/exo as control. Then, cellular RNA from GFP-S was extracted, and PCR was performed as described above. As an indirect measure of miRNA transfer, difference in *C*_t_ values between D/exo-treated GFP-S, and negative control at each time point were determined; positive values indicated miRNA transfer.

### Gene target prediction

The software PicTar (http://pictar.mdc-berlin.de/), MicroCosm (http://www.ebi.ac.uk/enright-srv/microcosm/htdocs/targets/v5/), and miRDB (http://www.mirdb.org/miRDB/) were applied to predict gene targets of selected miRNAs [18–20]. Only the genes listed by all these three independent tools were considered and searched for gene ontology (GO) term enrichment (http://www.geneontology.org) [21]. The Kyoto Encyclopedia of Genes and Genomes (KEGG) pathways were analyzed using the DAVID program (http://david.abcc.ncifcrf.gov/) [22,23].

### Statistical analysis

Data were analyzed using the SPSS 20.0 package, and graphs were generated by the GraphPad software. All experiments were performed in triplicate. Differences were determined by Student’s *t* test or by ANOVA, and *P*<0.05 was considered statistically significant.

## Results

### Exosome identification

**Figure 1:**
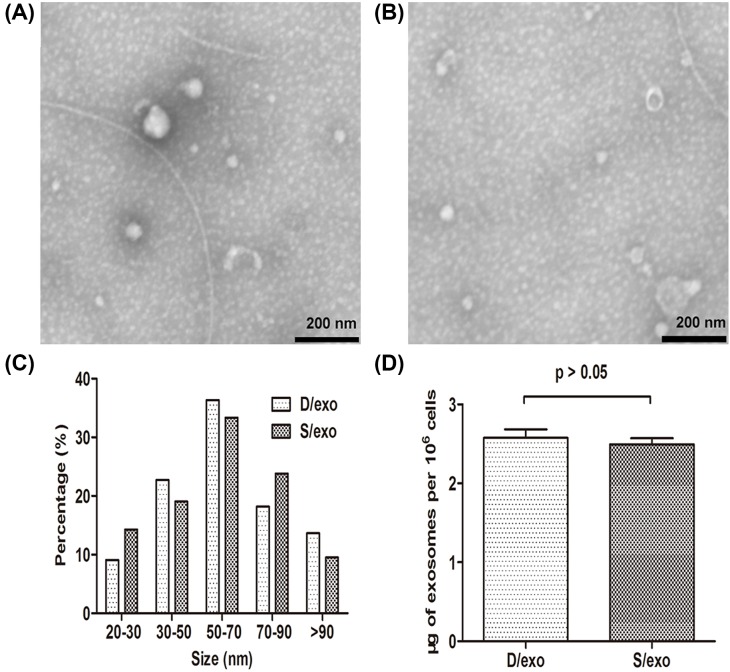
Exosome identification (**A**) Representative image of TEM of D/exo showing a spheroid shape (scale bar = 200 nm). (**B**) Representative image of TEM of S/exo showing a spheroid shape (scale bar = 200 nm). (**C**) D/exo and S/exo displayed a size ranging from 50 to 100 nm in diameter. (**D**) Exosome protein analysis indicated similar yield of D/exo and S/exo. Data are expressed as mean ± S.D., *n*=3.

After successful isolation of exosomes from the supernatants from MCF-7/Doc and MCF-7/S cells, we observed their morphology and size by TEM. D/exo and S/exo were homogeneous in appearance (Figure 1A,B), ranging from 50 to 100 nm in diameter, with a spheroid shape (Figure 1C). Besides, exosome quantities were similar (Figure 1D).

### D/exo transmit chemoresistance related to miRNAs

**Figure 2:**
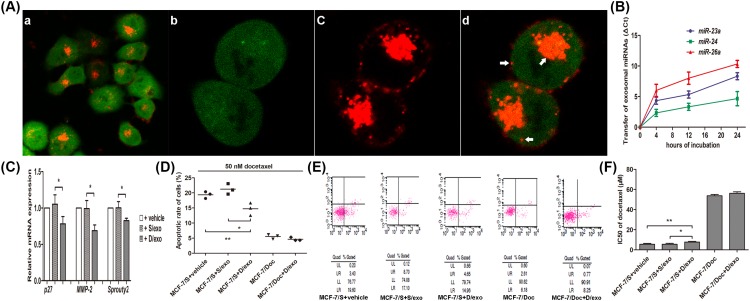
D/exo transmit chemoresistance (**A**) Confocal laser microscopy indicated successful uptake of D/exo by target GFP-S cells with which they were co-cultured. This image plate illustrates (a) absorption of PKH26-labeled D/exo by almost all the GFP-S at low magnification; (b) green fluorescence from GFP-S at high magnification; (c) PKH26 (red) staining of D/exo at high magnification; and (d) merge image at high magnification showing the incorporation and internalization of D/exo (arrows) on the cell membranes and inside cytoplasm of GFP-S. (**B**) GFP-S cells were incubated with D/exo for 4, 12, and 24 h, and transfer of miRNAs were analyzed by PCR. Time point 0 stands for GFP-S without D/exo. The difference in *C*_t_ values between the negative control (GFP-S alone) and D/exo-treated GFP-S is shown for each miRNA. Positive values indicate miRNA transfer. Data are expressed as mean ± S.D., *n*=3. (**C**) Relative expression of mRNAs in GFP-S was analyzed by PCR. Data are expressed as mean ± S.D., *n*=3, *P<0.05, +D/exo compared with +S/exo. (**D**) Evaluation of MCF-7/Doc incubated with D/exo, and MCF-7/S incubated with vehicle, S/exo, and D/exo for 72 h. Apoptotic rates of MCF-7/S and MCF-7/Doc were then determined after 24 h exposure to 50 nM docetaxel. Data are expressed as mean, *n*=3: *P<0.05, MCF-7/S+D/exo compared with MCF-7/S+S/exo. **P<0.05, MCF-7/S+D/exo compared with MCF-7/S+vehicle. (**E**) Representative scatter figures of apoptosis assay by flow cytometry. (**F**) Evaluation of MCF-7/Doc incubated with D/exo, and MCF-7/S incubated with vehicle, S/exo, and D/exo for 72 h. IC_50_ of docetaxel in MCF-7/S and MCF-7/Doc were then determined by MTT assay. Data are expressed as mean ± S.D., *n*=3: *P<0.05, MCF-7/S+D/exo compared with MCF-7/S+S/exo. **P<0.05, MCF-7/S+D/exo compared with MCF-7/S+vehicle.

To better visualize the uptake, GFP-S cells were employed and co-cultured with PKH26-labeled D/exo for 24 h, after which the absorption of exosomes by target cells was observed under a confocal microscope. At low magnification, we found that nearly all the GFP-S contained D/exo with red fluorescence (Figure 2A(a)). At high magnification, after merging green signals from two GFP cells (Figure 2A(b)) and red signals from stained D/exo (Figure 2A(c)), the incorporation and internalization of D/exo were indicated by several engulfed and diffused signals (arrows) on the cell membranes and inside cytoplasm (Figure 2A(d)). Consistent with D/exo uptake, we showed by comparing the variations in *C*_t_ values of miRNAs found in GFP-S alone with those of GFP-S incubated with D/exo that the abundance of several miRNAs (*miR-23a, miR-24*, and *miR-26a*) increased gradually (Figure 2B). Besides, D/exo treatment reduced mRNAs in GFP-S such as *Sprouty2, p27*, and *MMP-2*, known to be respectively targetted by the above miRNAs (Figure 2C).

To verify the potential of D/exo in resistance transmission, D/exo were collected and added into MCF-7/S cells. As expected, the apoptotic rate induced by 50 nM docetaxel was relatively high in MCF-7/S treated with vehicle. Incubation of MCF-7/S cells with D/exo decreased apoptotic rate, as compared with control cells treated with S/exo or vehicle, suggesting that D/exo induced apoptosis resistance (Figure 2D,E). Consistent with the data from apoptosis assay, we observed that IC_50_ of MCF-7/S treated with D/exo was higher with respect to control MCF-7/S incubated with S/exo or vehicle (Figure 2F). These results reinforced the significant role of D/exo and contained miRNAs in transferring chemoresistance to recipient cells.

### DRβ-H reduces exosome release

**Figure 3:**
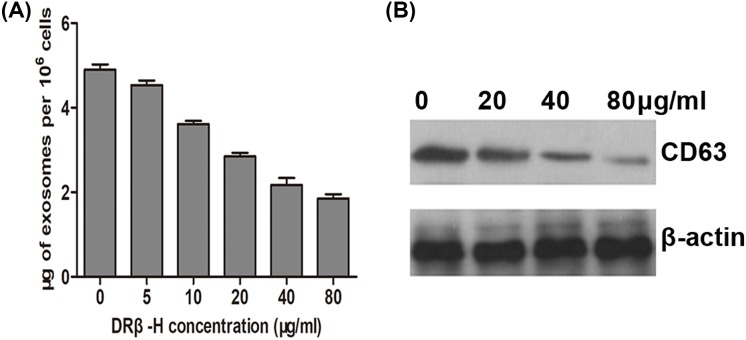
DRβ-H reduces exosome release (**A**) Exosome quantity of MCF-7/Doc treated by different concentrations of DRβ-H (0, 5, 10, 20, 40, and 80 μg/ml). Data are expressed as mean ± S.D., *n*=3. (**B**) Expression of exosomal marker CD63 in D/exo after MCF-7/Doc cells was treated by different concentrations of DRβ-H (0, 20, 40, and 80 μg/ml).

Our previous work reported the pro-apoptotic effects of DRβ-H on breast cancer cells and its comprehensive pathways [13]. Therefore, we wondered whether exosome secretion is involved in DRβ-H-mediated growth inhibition. We treated MCF-7/Doc cells with different concentrations of DRβ-H and found that D/exo secretion was decreased after DRβ-H treatment in a dose-dependent manner (Figure 3A). Besides, treatment with high concentration of DRβ-H significantly reduced exosomal marker CD63 with respect to control cells added with lower concentration of DRβ-H, further indicating that DRβ-H could inhibit D/exo release from MCF-7/Doc cells (Figure 3B).

### DRβ-H reverses chemoresistance related to exosomes

**Figure 4:**
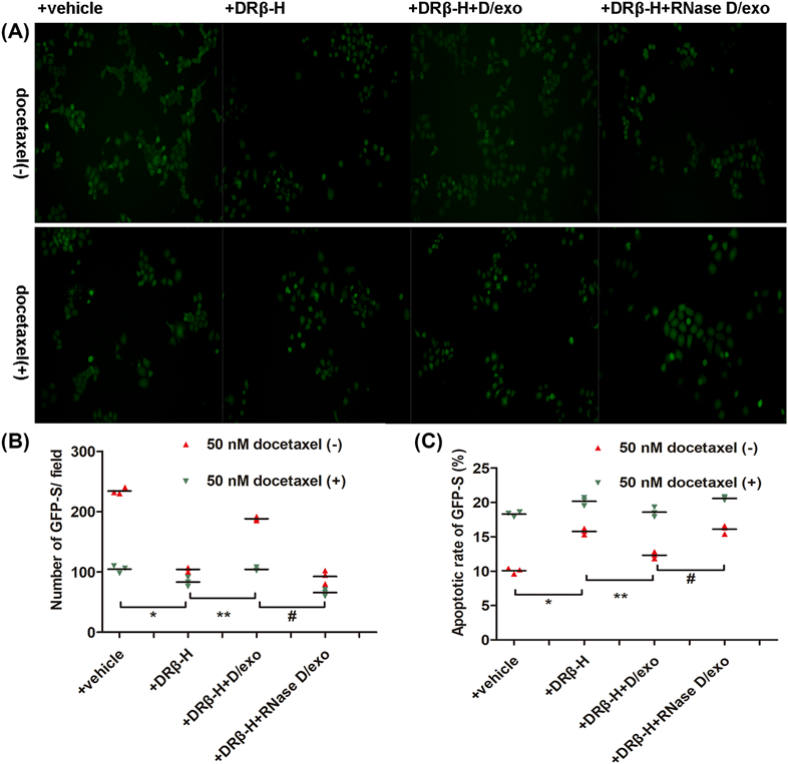
DRβ-H reverses chemoresistance related to exosomes Evaluation of GFP-S treated with or without 50 nM docetaxel after cell mixture was cultured with vehicle, DRβ-H, DRβ-H+D/exo, and DRβ-H+RNase D/exo. (**A**) Representative images showing the residual GFP-S cells treated under different conditions. (**B**) Quantitative evaluation of residual GFP-S. Data are expressed as mean ± S.D., *n*=3: *P<0.05, +DRβ-H compared with +vehicle. **P<0.05, +DRβ-H+D/exo compared with +DRβ-H. ^#^*P*<0.05, +DRβ-H+RNase D/exo compared with +DRβ-H+D/exo. (**C**) Apoptotic rate of residual GFP-S. Data are expressed as mean ± S.D., *n*=3: *P<0.05, +DRβ-H compared with +vehicle. **P<0.05, +DRβ-H+D/exo compared with +DRβ-H. ^#^*P*<0.05, +DRβ-H+RNase D/exo compared with +DRβ-H+D/exo.

We previously focussed on the function of exosomes moving from donor drug-resistant cells to recipient sensitive cells and confirmed the significance of D/exo from MCF-7/Doc as mediators to spread resistance capacity [9]. Here, we evaluated the role of exosomes in DRβ-H-mediated resistance reversal. As in our earlier publication, GFP-S cells were maintained with MCF-7/Doc at equal proportion under four conditions: (i) with vehicle, (ii) with DRβ-H, (iii) with DRβ-H+D/exo, and (iv) with DRβ-H+RNase D/exo, after which survival number and apoptotic rate of GFP-S were assessed in the presence of 50 nM docetaxel. Interestingly, incubation of cell mixture with DRβ-H resulted in significantly less surviving GFP-S after docetaxel exposure, as compared with those incubated with vehicle. Moreover, treatment of cell mixture with DRβ-H+D/exo increased GFP-S number with respect to control cells treated with DRβ-H only (Figure 4A,B). Likewise, the apoptotic rate was relatively high in cell mixture added with vehicle, whereas a marked increase in apoptotic rate was found in cell mixture added with DRβ-H. Treatment of cell mixture with DRβ-H+D/exo reduced apoptotic rate with respect to control cells treated with DRβ-H only (Figure 4C). These data collectively suggested that DRβ-H could reverse chemoresistance and that such effect could be partly attributed to D/exo.

RNase pretreatment of D/exo (referred to as RNase D/exo) significantly reduced surviving GFP-S cells and conferred apoptosis resistance, with respect to D/exo, reinforced the role of RNA molecules loaded by D/exo (Figure 4A–C).

### DRβ-H regulates exosomal miRNA expression

**Figure 5:**
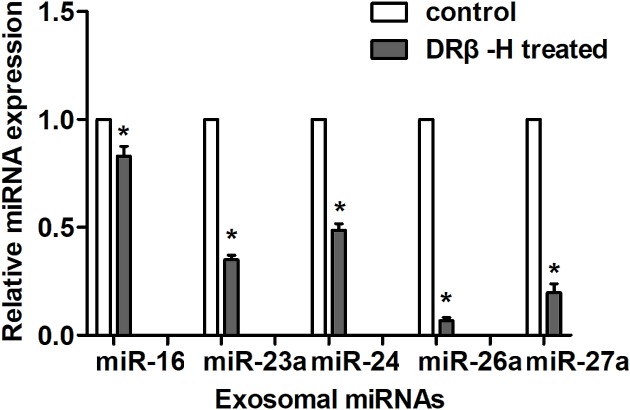
DRβ-H regulates exosomal miRNA expression Relative expressions of selected exosomal miRNAs in D/exo after MCF-7/Doc were treated with or without DRβ-H. Data are expressed as mean ± S.D., *n*=3: *P<0.05, DRβ-H treated compared with control.

We recently analyzed the miRNA profiles of D/exo and MCF-7/Doc by microarray and demonstrated that the top 20 most abundant miRNAs transported by D/exo (Table 1) were involved in signaling pathways related to therapy failure [9]. To test the hypothesis that DRβ-H could regulate exosomal miRNA expression, we picked five miRNAs (*miR-16, miR-23a, miR-24, miR-26a*, and *miR-27a*) from the top 20 exosomal miRNAs and checked their levels after cells were exposed to DRβ-H. In particular, the treatment of MCF-7/Doc with DRβ-H resulted in less expression of all the selected miRNAs in the corresponding D/exo, as compared with those expressed in original D/exo (Figure 5).

**Table 1:** The top 20 most abundant miRNAs transported by D/exo

miRNA ID	Log_2_ (fold change)*	miRNA ID	Log_2_ (fold change)
hsa-*miR-23a*	9.88	hsa-*miR-15b*	5.96
hsa-*miR-1246*	9.60	hsa-*miR-1290*	5.72
hsa-let-7b	9.01	hsa-*miR-31*	5.70
hsa-let-7a	8.50	hsa-*miR-92a*	5.25
hsa-*miR-16*	8.13	hsa-*miR-191*	5.16
hsa-let-7c	8.04	hsa-*miR-30c*	4.62
hsa-*miR-23b*	7.72	hsa-*miR-182*	4.31
hsa-*miR-27a*	7.39	hsa-*miR-3188*	4.20
hsa-*miR-103a*	6.83	hsa-*miR-425*	3.60
hsa-*miR-26a*	6.58	hsa-*miR-24*	3.06

*Fold change = (D/exo)/(S/exo)

To understand the biological processes influenced by the upstream differentially expressed miRNAs, we predicted their targets by three independent algorithms, PicTar, MicroCosm, and miRDB. Only the genes listed by all these tools were taken into account. We totally detected 293 putative genes and then performed a GO enrichment analysis using DAVID programme. As listed in Table 2, we found a strong over-representation of terms belonging to crucial biological processes. According to KEGG analysis, the candidate genes were suggested to participate in several signaling pathways such as cell cycle, Wnt signaling pathway, focal adhesion, and mRNA surveillance pathway (Table 3).

**Table 2:** GO term analysis with DAVID tool

GO ID	GO term	Gene count	%	*P*-value
GO:0005654	Nucleoplasm	67	24.0	5.0E-5
GO:0005737	Cytoplasm	107	38.4	1.0E-4
GO:0043235	Receptor complex	10	3.6	1.2E-4
GO:0005829	Cytosol	73	26.2	3.4E-4
GO:0005634	Nucleus	106	38.0	7.2E-4
GO:0043231	Membrane-bounded organelle	18	6.5	4.1E-3
GO:1990909	Wnt signalosome	3	1.1	1.1E-2
GO:0031672	A band	3	1.1	1.8E-2
GO:0030877	β-catenin destruction complex	3	1.1	1.8E-2
GO:0005874	Microtubule	11	3.9	1.8E-2
GO:0005769	Early endosome	9	3.2	2.1E-2
GO:0048471	Perinuclear region of cytoplasm	17	6.1	2.3E-2
GO:0016581	NuRD complex	3	1.1	2.9E-2
GO:0000785	Chromatin	5	1.8	4.3E-2
GO:0034673	Inhibin–β-glycan–ActRII complex	2	0.7	4.4E-2
GO:0036464	Cytoplasmic ribonucleoprotein granule	3	1.1	5.2E-2
GO:1990851	Wnt–Frizzled–LRP5/6 complex	2	0.7	5.8E-2
GO:0005730	Nucleolus	19	6.8	8.7E-2
GO:0072357	PTW/PP1 phosphatase complex	2	0.7	9.9E-2

**Table 3:** KEGG pathway analysis with DAVID tool

Pathway ID	Pathway term	Genes	%	*P*-value
has04510	Focal adhesion	*CRKL, RAP1A, COL1A2, COL11A2, GSK3B, PPP1CB, PPP1R12A, RELN, VAV1, ZYX*	3.6	1.0E-2
has03015	mRNA surveillance pathway	*GSPT1, PYM1, NXF1, NXT2, PPP1CB, PPP2R5E*	2.2	2.2E-2
has04310	Wnt signaling pathway	*LRP5, LRP6, WNT7A, CTNNBIP1, GSK3B, NFATC3, SIAH1*	2.5	3.5E-2
has04931	Insulin resistance	*GFPT2, GSK3B, INSR, PRKCD, PPP1CB, RPS6KA6*	2.2	4.2E-2
has04722	Neurotrophin signaling pathway	*CRKL, RAP1A, BEX3, GSK3B, PRKCD, RPS6KA6*	2.2	6.1E-2
has04110	Cell cycle	*WEE1, CHEK1, CCNE1, CCNH, GSK3B, YWHAQ*	2.2	6.8E-2

## Discussion

Docetaxel-based chemotherapy plays a vital role against breast cancer, but the efficacy is mainly restricted by drug resistance. Previously, we reported that DRβ-H, a novel component obtained from natural plant *C. ganpiniana*, exerts strong inhibitory activity on different breast cancer cells and may be a potential option for drug development and tumor treatment [12–14]. In the present work, we first report that DRβ-H could suppress breast cancer growth by regulating intercellular resistance transmission via reducing exosome release.

The emerging evidence that cells may communicate with surrounding cancer cells or stromal cells within tumor microenvironment through the secretion of exosomes carrying genetic information has recently highlighted the importance of these entities [2,3,10,24]. In the present study, D/exo from MCF-7/Doc could be absorbed by recipient breast cancer cells, and are effective in transferring chemoresistance from drug-resistant cells to sensitive ones. This was based on the confocal microscopic visualization of red-labeled D/exo on the cell membranes and inside cytoplasm of GFP-S and further verified by analyzing the apoptosis and MTT assay. We also confirmed that miRNAs delivered by D/exo were functional because able to reduce the target gene expressions. A similar pattern was found in prostate cancer cells to docetaxel and lung cancer cells to cisplatin, reinforcing the potential of tumor-derived exosomes in resistance transmission [6,25].

Recently, the anticancer effects of DRβ-H have attracted much attention. Our previous study provided apoptotic activity and the mechanism for the anticancer capacity of DRβ-H [13]; however, the relevance of exosomes during DRβ-H exposure has yet to be determined. In the present study, D/exo yield was decreased after DRβ-H treatment in a dose-dependent manner, and exosomal marker CD63 was negatively correlated with DRβ-H concentration, indicating that DRβ-H could influence D/exo release from MCF-7/Doc cells. Several mechanisms have been suggested to control the biogenesis and shedding of exosomes [24]. Although the underlying molecular machinery remains largely unclear, it seems plausible that reducing the formation and secretion of exosomes may be a novel therapeutic strategy [10,11]. Besides, many specific exosomal markers including CD63 could be found in the popular exosome database Exocarta (http://www.exocarta.org), offering more methods for exosome detection [26].

Limited studies, to date, have demonstrated that exosomes were involved in reversing drug insensitivity, we therefore evaluated the role of exosomes in DRβ-H-mediated resistance reversal. Interestingly we found reduced docetaxel resistance in cell mixture in the presence of DRβ-H, compared with when these cells were treated with vehicle. In keeping with the findings from our initial exosome co-culture assay, we observed a similar trend of conferred docetaxel resistance to cell mixture in the presence of DRβ-H and D/exo, with respect to cell mixture treated with DRβ-H only. These indicated that DRβ-H could reverse chemoresistance and that such effect could be partly attributed to D/exo. Thus, it is speculated that drug-resistant breast cancer cells could spread resistance traits to residual sensitive cells via exosomes after toxic insult, whereas DRβ-H blocks resistance transmission by reducing exosomes from drug-resistant cells. We have not tested the efficacy of DRβ-H and D/exo in reversing drug sensitivity, further investigations are needed to more precisely elucidate the relevance.

Remarkably, an explanation for the manner whereby D/exo restored docetaxel resistance of cell mixture in the presence of DRβ-H is that they are transferring miRNAs from MCF-7/Doc that are causal molecules in changing the susceptibility of co-cultured GFP-S. Our study suggests that the miRNA content of D/exo plays an important role, as the RNase treatment of D/exo significantly abrogated the biological effects of D/exo. Although we have not applied the miRNA microarray method to detect all the differentially expressed exosomal miRNAs after DRβ-H treatment, we showed that DRβ-H influenced the expressions of all the selected exosomal miRNAs. Based on target prediction and pathway analysis, these miRNAs were suggested to participate in pathways related to treatment failure, such as cell cycle, Wnt signaling pathway, focal adhesion, and mRNA surveillance pathway [27,28]. This is in keeping with our previous publication that the top 20 most abundant miRNAs transported by D/exo take part in signaling pathways related to therapy failure [9].

In conclusion, the results of the present study expand on previous findings and provide the first evidence suggesting that, in breast cancer, DRβ-H acts through reducing the secretion of exosomes from drug resistant cells and that this mechanism may be responsible for DRβ-H-mediated resistance reversal. This would offer new insights into design new strategy for overcoming exosome-associated resistance transmission.

## Abbreviations

DRβ-H: drhamnose β-hederin
GFP-S: MCF-7/S expressing GFP
GO: gene ontology
KEGG: Kyoto Encyclopedia of Genes and Genomes
MCF-7/Doc: docetaxel-resistant breast cancer cell MCF-7

## References

[1] DeSantis C.E., Ma J., Goding Sauer A., Newman L.A. and Jemal A. (2017) Breast cancer statistics, 2017, racial disparity in mortality by state. CA Cancer J. Clin. 67, 439–448 28972651 10.3322/caac.21412

[2] Chin A.R. and Wang S.E. (2016) Cancer-derived extracellular vesicles: the ‘soil conditioner’ in breast cancer metastasis? Cancer Metastasis Rev. 35, 669–676 10.1007/s10555-016-9639-8 27838868 PMC5831362

[3] Chen W.X., Zhong S.L., Ji M.H. (2014) MicroRNAs delivered by extracellular vesicles: an emerging resistance mechanism for breast cancer. Tumour Biol. 35, 2883–2892 10.1007/s13277-013-1417-4 24272085

[4] Valadi H., Ekstrom K., Bossios A., Sjostrand M., Lee J.J. and Lotvall J.O. (2007) Exosome-mediated transfer of mRNAs and microRNAs is a novel mechanism of genetic exchange between cells. Nat. Cell Biol. 9, 654–659 10.1038/ncb1596 17486113

[5] Yu X., Odenthal M. and Fries J.W. (2016) Exosomes as miRNA carriers: formation-function-future. Int. J. Mol. Sci. 17, pii: E2028 10.3390/ijms17122028 PMC5187828 27918449

[6] Corcoran C., Rani S., O’Brien K. (2012) Docetaxel-resistance in prostate cancer: evaluating associated phenotypic changes and potential for resistance transfer via exosomes. PLoS ONE 7, e50999 10.1371/journal.pone.0050999 23251413 PMC3519481

[7] Skog J., Wurdinger T., van Rijn S. (2008) Glioblastoma microvesicles transport RNA and proteins that promote tumour growth and provide diagnostic biomarkers. Nat. Cell Biol. 10, 1470–1476 10.1038/ncb1800 19011622 PMC3423894

[8] Kogure T., Lin W.L., Yan I.K., Braconi C. and Patel T. (2011) Intercellular nanovesicle-mediated microRNA transfer: a mechanism of environmental modulation of hepatocellular cancer cell growth. Hepatology 54, 1237–1248 10.1002/hep.24504 21721029 PMC3310362

[9] Chen W.X., Cai Y.Q., Lv M.M. (2014) Exosomes from docetaxel-resistant breast cancer cells alter chemosensitivity by delivering microRNAs. Tumour Biol. 35, 9649–9659 10.1007/s13277-014-2242-0 24969560

[10] Kourembanas S. (2015) Exosomes: vehicles of intercellular signaling, biomarkers, and vectors of cell therapy. Annu. Rev. Physiol. 77, 13–27 10.1146/annurev-physiol-021014-071641 25293529

[11] Tkach M. and Thery C. (2016) Communication by extracellular vesicles: where we are and where we need to go. Cell 164, 1226–1232 10.1016/j.cell.2016.01.043 26967288

[12] Ding Q., Yang L.X., Yang H.W., Jiang C., Wang Y.F. and Wang S. (2009) Cytotoxic and antibacterial triterpenoids derivatives from *Clematis ganpiniana*. J. Ethnopharmacol. 126, 382–385 10.1016/j.jep.2009.09.028 19781611

[13] Cheng L., Xia T.S., Wang Y.F. (2014) The apoptotic effect of D Rhamnose beta-hederin, a novel oleanane-type triterpenoid saponin on breast cancer cells. PLoS ONE 9, e90848 10.1371/journal.pone.0090848 24603880 PMC3946269

[14] Cheng L., Xia T.S., Shi L. (2018) D Rhamnose beta-hederin inhibits migration and invasion of human breast cancer cell line MDA-MB-231. Biochem. Biophys. Res. Commun. 495, 775–780 10.1016/j.bbrc.2017.11.081 29146183

[15] Hanahan D. and Weinberg R.A. (2011) Hallmarks of cancer: the next generation. Cell 144, 646–674 10.1016/j.cell.2011.02.013 21376230

[16] Zhong S., Li W., Chen Z., Xu J. and Zhao J. (2013) MiR-222 and miR-29a contribute to the drug-resistance of breast cancer cells. Gene 531, 8–14 10.1016/j.gene.2013.08.062 23994196

[17] Chen W.X., Liu X.M., Lv M.M. (2014) Exosomes from drug-resistant breast cancer cells transmit chemoresistance by a horizontal transfer of microRNAs. PLoS ONE 9, e95240 10.1371/journal.pone.0095240 24740415 PMC3989268

[18] Krek A., Grun D., Poy M.N. (2005) Combinatorial microRNA target predictions. Nat. Genet. 37, 495–500 10.1038/ng1536 15806104

[19] Griffiths-Jones S., Saini H.K., van Dongen S. and Enright A.J. (2008) miRBase: tools for microRNA genomics. Nucleic Acids Res. 36, D154–158 10.1093/nar/gkm952 17991681 PMC2238936

[20] Wong N. and Wang X. (2015) miRDB: an online resource for microRNA target prediction and functional annotations. Nucleic Acids Res. 43, D146–152 10.1093/nar/gku1104 25378301 PMC4383922

[21] Ashburner M., Ball C.A., Blake J.A. (2000) Gene ontology: tool for the unification of biology. The gene ontology consortium. Nat. Genet. 25, 25–29 10.1038/75556 10802651 PMC3037419

[22] Kanehisa M., Araki M., Goto S. (2008) KEGG for linking genomes to life and the environment. Nucleic Acids Res. 36, D480–D484 10.1093/nar/gkm882 18077471 PMC2238879

[23] Huang D.W., Sherman B.T., Tan Q. (2007) DAVID Bioinformatics Resources: expanded annotation database and novel algorithms to better extract biology from large gene lists. Nucleic Acids Res. 35, W169–W175 10.1093/nar/gkm415 17576678 PMC1933169

[24] Chen X., Liang H., Zhang J., Zen K. and Zhang C.Y. (2012) Secreted microRNAs: a new form of intercellular communication. Trends Cell Biol. 22, 125–132 10.1016/j.tcb.2011.12.001 22260888

[25] Xiao X., Yu S., Li S. (2014) Exosomes: decreased sensitivity of lung cancer A549 cells to cisplatin. PLoS ONE 9, e89534 10.1371/journal.pone.0089534 24586853 PMC3931805

[26] Mathivanan S., Fahner C.J., Reid G.E. and Simpson R.J. (2012) ExoCarta 2012: database of exosomal proteins, RNA and lipids. Nucleic Acids Res. 40, D1241–D1244 10.1093/nar/gkr828 21989406 PMC3245025

[27] Glackin C.A. (2014) Targeting the Twist and Wnt signaling pathways in metastatic breast cancer. Maturitas 79, 48–51 10.1016/j.maturitas.2014.06.015 25086726

[28] Wu X., Zahari M.S., Renuse S. (2015) Phosphoproteomic analysis identifies focal adhesion kinase 2 (FAK2) as a potential therapeutic target for tamoxifen resistance in breast cancer. Mol. Cell. Proteomics 14, 2887–2900 10.1074/mcp.M115.050484 26330541 PMC4638033

